# RSAD2 suppresses viral replication by interacting with the Senecavirus A 2 C protein

**DOI:** 10.1186/s13567-024-01370-2

**Published:** 2024-09-27

**Authors:** Lei Hou, Zhi Wu, Penghui Zeng, Xiaoyu Yang, Yongyan Shi, Jinshuo Guo, Jianwei Zhou, Jiangwei Song, Jue Liu

**Affiliations:** 1 College of Veterinary Medicine, Yangzhou University, Yangzhou, China; 2 Jiangsu Co-Innovation Center for Prevention and Control of Important Animal Infectious Diseases and Zoonoses, Yangzhou University, Yangzhou, China; 3 Loudi Livestock, Aquaculture, and Agricultural Machinery Affairs Center, Loudi, China; 4 Beijing Key Laboratory for Prevention and Control of Infectious Diseases in Livestock and Poultry, Institute of Animal Husbandry and Veterinary Medicine, Beijing Academy of Agriculture and Forestry Sciences, Beijing, China

**Keywords:** RSAD2, SVA replication, interaction, 2 C protein, JAK-STAT pathway

## Abstract

Senecavirus A (SVA), an emerging virus that causes blisters on the nose and hooves, reduces the production performance of pigs. RSAD2 is a radical S-adenosylmethionine (SAM) enzyme, and its expression can suppress various viruses due to its broad antiviral activity. However, the regulatory relationship between SVA and RSAD2 and the mechanism of action remain unclear. Here, we demonstrated that SVA infection increased *RSAD2* mRNA levels, whereas RSAD2 expression negatively regulated viral replication, as evidenced by decreased viral VP1 protein expression, viral titres, and infected cell numbers. Viral proteins that interact with RSAD2 were screened, and the interaction between the 2 C protein and RSAD2 was found to be stronger than that between other proteins. Additionally, amino acids (aa) 43–70 of RSAD2 were crucial for interacting with the 2 C protein and played an important role in its anti-SVA activity. RSAD2 was induced by type I interferon (IFN-I) via Janus kinase signal transducer and activator of transcription (JAK-STAT), and had antiviral activity. Ruxolitinib, a JAK-STAT pathway inhibitor, and the knockdown of JAK1 expression substantially reduced RSAD2 expression levels and antiviral activity. Taken together, these results revealed that RSAD2 blocked SVA infection by interacting with the viral 2 C protein and provide a strategy for preventing and controlling SVA infection.

## Introduction

Senecavirus A (SVA), previously known as Seneca Valley virus (SVV), is a non-enveloped virus with a positive-sense single-stranded RNA genome that belongs to the genus *Senecavirus* of the *Picornaviridae* family [1]. SVA was first isolated from PER.C6 cell culture media, possibly containing contaminated porcine trypsin or foetal bovine serum [1]. Since 2014, vesicular disease outbreaks triggered by SVA have occurred in many countries [2]. SVA infections were first reported in China in 2015 [3], and many SVA strains are prevalent in pig herds.

The full-length SVA genome contains a long open reading frame (ORF) encoding a single polyprotein. This polyprotein is processed into structural and non-structural proteins by viral and host proteases [1]. The 2 C protein, one of the most conserved non-structural proteins in picornaviruses, possesses ATPase and helicase activities and is involved in viral replication [4–6]. It is classified as a typical member of the SF3 helicases on the basis of conserved motifs [5]. The homo-oligomeric form of the 2 C protein in picornaviruses is required for their biological activity [7]. SVA 2 C has developed various strategies for facilitating viral replication. It reduces retinoic acid-inducible gene (RIG-I) and cyclic GMP-AMP synthase (cGAS) levels through the caspase and autophagy pathways, respectively; inhibits IFN production; and impairs host innate immunity [8, 9]. The SVA 2 C protein induces apoptosis via a mitochondria-mediated intrinsic pathway [10].

Radical S-adenosyl methionine domain–containing 2 (RSAD2), also known as viperin, is an IFN-stimulated gene (ISG) that can be induced in diverse cell types by IFN-α and IFN-β [11, 12] and by infection with various viruses [12, 13]. The structure of RSAD2 is divided into an N-terminal dual alpha-helical domain (aa 1–71), an intermediate S-adenosylmethionine (SAM) domain (aa 72–182), and a C-terminal domain (aa 183–361) [14]. The N-terminal domain of RSAD2 (aa 1–42) is associated with localization to the endoplasmic reticulum (ER) and cell membrane lipid raft structures, whereas the SAM domain is responsible for RNA synthesis [15]. For example, the colocalization of the RSAD2 N-terminal domain and the hepatitis C virus NS5A protein in lipid droplets blocks NS5A binding to other proteins and impairs viral replication [16]. A specific motif in the SAM domain of RSAD2 regulates tick-borne encephalitis virus (TBEV) RNA synthesis by binding to iron-sulfur clusters [15]. In addition, the C-terminal domain contains a highly conserved region that is associated with RSAD2 antiviral activity [17]. Several studies have shown that RSAD2 has diverse antiviral mechanisms and suppresses picornavirus replication. RSAD2 modulates coxsackievirus A16 replication, possibly by associating with viral 5′ untranslated regions [18]. RSAD2 inhibits enterovirus A71 replication by interacting with viral 2 C proteins [19]. However, the effects of RSAD2 on SVA replication and the underlying regulatory mechanisms remain unknown.

In this study, we assessed the role of RSAD2 in SVA replication and revealed that a reduction in the RSAD2 expression level was associated with active SVA infection and that its expression negatively regulated SVA replication. We further screened the viral protein(s) responsible for the interaction with RSAD2 and identified an interaction between the SVA 2 C protein and RSAD2, with aa 43–70 of the RSAD2 N-terminal domain being important for this interaction and antiviral activity. Additionally, we confirmed that RSAD2, an interferon (IFN)-stimulated gene (ISG), is involved in IFN-mediated anti-SVA function in host cells treated with an IFN inducer, a JAK-STAT pathway inhibitor, and a siRNA targeting *JAK1*. These results clarify the mechanism of RSAD2-mediated antiviral activity and identify important targets for preventing and controlling SVA replication.

## Materials and methods

### Cells, viruses, and antibodies

PK-15 cells, HEK-293T cells, and BHK-21 cells were originally obtained from the American Type Culture Collection and cultured in Dulbecco’s modified Eagle’s medium (Gibco, Waltham, MA, USA) containing 5–10% foetal bovine serum (FBS; Gibco, Life Technologies), streptomycin, and penicillin at 37 °C in a 5% CO_2_ incubator. The SVA CHhb17 strain and Sendai virus (SeV) preserved in our laboratory were used in this study. An anti-SVA VP1 monoclonal antibody was obtained from our laboratory, and other antibodies were purchased from commercial suppliers. These included rabbit anti-Flag (0912-1; HuaAn), rabbit anti-GFP (ET1602; HuaAn), mouse anti-β-actin (D191047; Sangon), rabbit anti-RSAD2 (A8271, ABclonal), and rabbit anti-JAK1 (A5534, ABclonal) antibodies and horseradish peroxidase (HRP)-conjugated anti-rabbit or anti-mouse (A0545 or A9044; Sigma‒Aldrich) and tetramethylrhodamine isothiocyanate–conjugated anti-rabbit secondary antibodies (Abcam, ab6799).

### Chemical reagents

Ruxolitinib (SD4740) was obtained from Beyotime (China), and 4′,6′-diamidino-2-phenylindole (DAPI; D8417) was obtained from Sigma‒Aldrich. Poly(I: C) (HY-107202; MedChemExpress) was dissolved in dimethyl sulfoxide or ultrapure water in accordance with the manufacturer’s recommendations.

### Plasmid construction and transfection

All the plasmids encoding various viral proteins used in this study were stored in our laboratory. The cDNA of porcine *RSAD2* (access number: NM_213817) was obtained by reverse-transcription polymerase chain reaction (RT‒PCR) and then subcloned and inserted into p3×Flag plasmids. All the generated mutant plasmids of the *RSAD2* and viral *2 C* genes were corrected by sequencing. All the primers used for plasmid construction are listed in Table 1.

BHK-21 or PK-15 cells cultured in monolayers to approximately 70–80% confluence were transfected with the indicated plasmids using Lipofectamine 2000 (11668019, Invitrogen) in accordance with the manufacturer’s protocol. After infection with SVA, the cells were analysed at the indicated time points.

**Table 1:** **Primers and corresponding sequences**

Primers	Sequence (5′-3′)
pEGFP-2C (1-200 aa)-F	GGTACCGGGACCCATGGATACAGTCAAAG
pEGFP-2C (1-200 aa)-R	GGATCCACGCCATGTTGGGAAGAAATTG
pEGFP-2C (101-322 aa)-F	GGTACCGACCACTATGATCAATGCCAAG
pEGFP-2C (101-322 aa)-R	GGATCCACTGTAGAACCAGAGTCTGC
pEGFP-2C (201-322 aa)-F	GGTACCGGCCCTTGCAGAGAAGGGGC
pEGFP-2C (201-322 aa)-R	GGATCCACTGTAGAACCAGAGTCTGC
p3×Flag-RSAD2-ΔN-F	GCGGCCGCGAATTCAATGACCACCCCCACT
p3×Flag-RSAD2-ΔN-R	AGTGGGGGTGGTCATTGAATTCGCGGCCGC
p3×Flag-RSAD2-ΔSAM-F	GACAGCCATCTGCCCAGCTTTGATGAGCAG
p3×Flag-RSAD2-ΔSAM-R	CTGCTCATCAAAGCTGGGCAGATGGCTGTC
p3×Flag-RSAD2-ΔC-F	ATCTCCTGTGACAGCTGATCGGTACCAGTC
p3×Flag-RSAD2-ΔC-R	GACTGGTACCGATCAGCTGTCACAGGAGAT
p3×Flag-RSAD2-Δ (43-70 aa)-F	GCTTTCTGGCGGGCAACCACCCCCACTAGC
p3×Flag-RSAD2-Δ (43-70 aa)-R	GCTAGTGGGGGTGGTTGCCCGCCAGAAAGC
p3×Flag-RSAD2-ΔERLD-F	GCCGCGAATTCAATGGGGGGTGATAGGAGC
p3×Flag-RSAD2-ΔERLD-R	GCTCCTATCACCCCCCATTGAATTCGCGGC
p3×Flag-RSAD2-F	GCGAATTCATGTGGACACTGGTACCTG
p3×Flag-RSAD2-R	CTGGTACCGATCACCAGTCCAGCTTC

### Coimmunoprecipitation (co-IP) and western blotting

HEK-293T cells co-transfected with pEGFP-L, pEGFP-VP3, pEGFP-VP4, pEGFP-2 C pEGFP-3 C, or pEGFP-C1 plasmids and/or p3×Flag-RSAD2, p3×Flag-RSAD2 mutants, or p3×Flag plasmids for 24 h were lysed with NP40 lysis buffer containing phenylmethylsulfonyl fluoride (ST506, Beyotime), and the supernatants were immunoprecipitated with anti-GFP agarose (PGA025, Lablead) or anti-Flag agarose (PFA025, Lablead) at 4 °C on a roller. After five washes, the immunoprecipitates were harvested and analysed. For western blotting, the quantified cell lysates were analysed by sodium dodecyl sulfate‒polyacrylamide gel electrophoresis, followed by transfer onto a nitrocellulose (NC) membrane (66485; Pall). The membranes were blocked with 5% non-fat milk and incubated with the appropriate primary and secondary antibodies. The NC membranes were exposed using a SuperSignal West Pico PLUS Chemiluminescent Substrate Kit (34580; Thermo) in an AMERSHAM ImageQuant800 chemiluminescence imaging system (GE, USA).

### Viral infection and 50% tissue culture infectious dose (TCID50) assay

After being washed with phosphate-buffered saline (PBS), the cells were incubated with SVA (multiplicity of infection (MOI) = 1) for 1 h at 37 °C. After the unbound virus was removed, the infected cells were maintained in medium supplemented with 2% FBS for the indicated time points and then collected and assayed for viral titres by serial dilution. PK-15 and BHK-21 cells were seeded into 96-well cell culture plates. The monolayer cells were inoculated with 100 µL of 10-fold serial dilutions of samples and were tested in eight replicates. The cells were cultured until cytopathic effects were observed. TCID_50_ values were determined using Spearman and Karber’s method.

### RNA extraction and reverse transcription quantitative polymerase chain reaction (RT-qPCR)

Total RNA was extracted from the samples using TRIzol reagent (15596018; Invitrogen) and cDNA was synthesized using the Vazyme cDNA Synthesis Kit (R323-01; Vazyme). cDNA samples were amplified using the Taq Pro Universal SYBR qPCR Master Mix Kit (Q712-02; Vazyme). The glyceraldehyde-3-phosphate dehydrogenase (*GAPDH*) gene was used as the internal control. The relative levels of mRNA were calculated via the comparative cycle threshold (2^−ΔΔCT^) method. The sequences of primers used were as follows: *GAPDH* (porcine)-F (TCGGAGTGAACGGATTTGGC) and *GAPDH* (porcine)-R (TGACAAGCTTCCCGTTCTCC), *RSAD2* (porcine)-F: AGTGTCAGCATCGTGAGCAA and *RSAD2* (porcine)-R: AAGCTGTCACAGGAGATGGC, *IFN-β* (porcine)-F: ACCAACAAAGGAGCAG and *IFN-β* (porcine)-R: TTTCATTCCAGCCAGT, *GAPDH* (hamster)-F: GTCATCATCTCCGCCCCTTC and *GAPDH* (hamster)-R: CCGTGGTCATGAGTCCTTCC, and *RSAD2* (hamster)-F: CGTGAGCATCGTGAGCAATG and *RSAD2* (hamster)-R: TGCACCACTTCCTCAGCTTT.

### RNA interference

PK-15 and BHK-21 cells were transfected with siRNA targeting RSAD2 (sc-94261; Santa Cruz) or JAK1 (sc-35719; Santa Cruz) or control siRNA (siCon) for 36 h according to the manufacturer’s protocol, infected with SVA, and then processed and analysed by western blotting and TCID_50_.

### Immunofluorescence assays and confocal microscopy

BHK-21 cells grown to approximately 80–90% confluence were transfected with the indicated plasmids or infected with SVA (GFP) at different time points in 24-well culture plates, followed by fixation with 4% paraformaldehyde for 20 min and permeabilization with 0.1% Triton X-100 at room temperature for 10 min. After washing with PBS, the cells were blocked with 5% non-fat milk, followed by incubation with appropriate primary and secondary antibodies and DAPI. Images were then obtained using an immunofluorescence microscope (IX73; Olympus, Tokyo, Japan) or a confocal immunofluorescence microscope (TCS SP8 STED; Leica, Weztlar, Germany).

### Cell viability assay

The effects of the chemical reagents on cell viability were detected using an MTT cell proliferation and cytotoxicity assay kit (C0009M; Beyotime). PK-15 cells were treated with various concentrations of chemical reagents, and cell viability was measured at the indicated time points in accordance with the manufacturer’s protocol.

### Statistical analysis

Significant differences were evaluated using one-way analysis of variance (ANOVA) or Student’s *t* test using Prism 9.0 software (GraphPad Software), with a *P* value < 0.05 considered statistically significant.

## Results

### SVA infection reduces RSAD2 expression in BHK-21 and PK-15 cells

**Figure 1:**
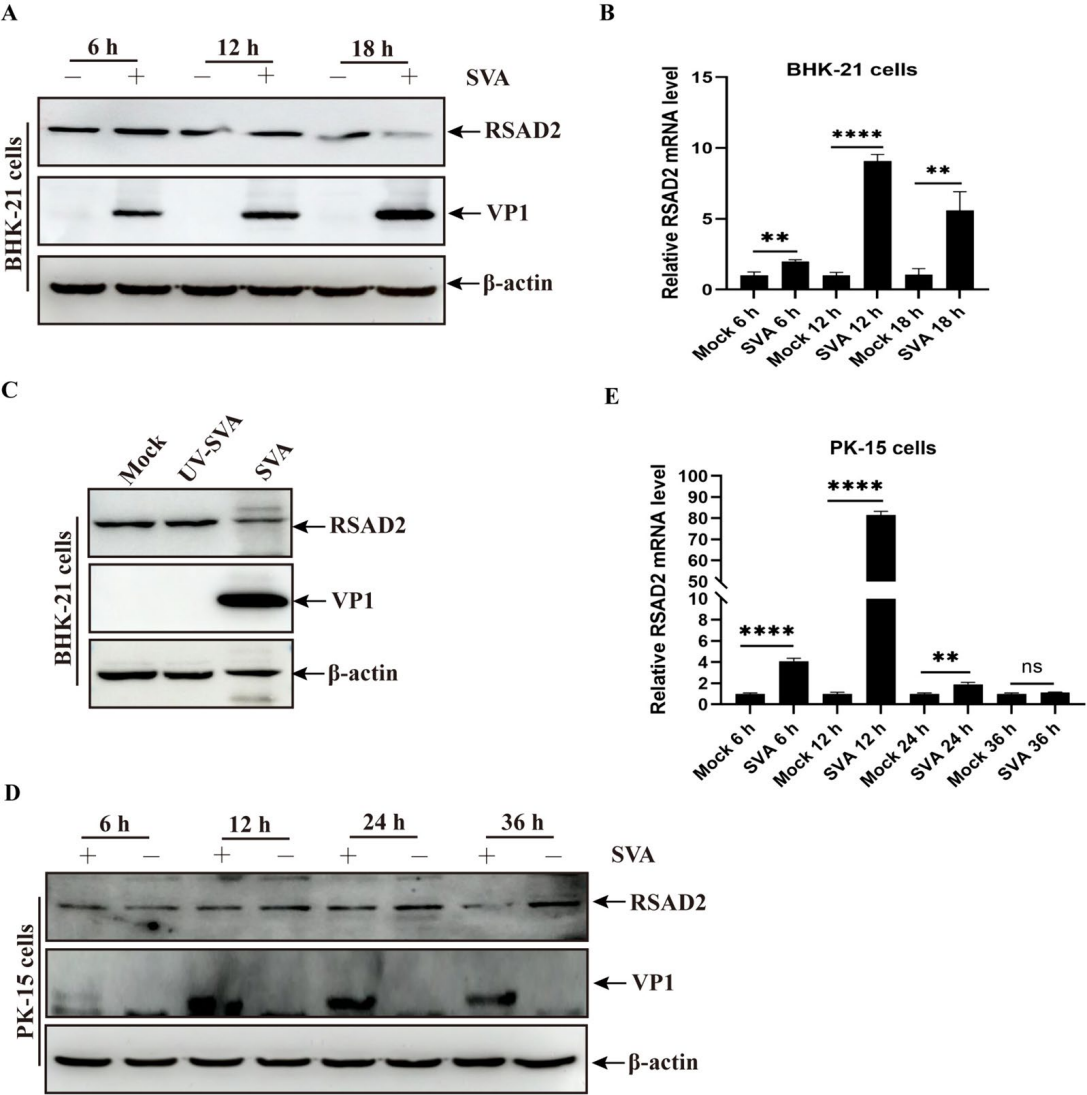
**SVA infection reduced the expression levels of RSAD2 in BHK-21 and PK-15 cells. A** and **B** The extracted proteins and *RSAD2* mRNA from SVA- or mock-infected BHK-21 cells at various time points were analysed by western blotting with anti-RSAD2, anti-VP1, and anti-β-actin antibodies (**A**) and reverse transcription‒quantitative polymerase chain reaction (RT-qPCR) (**B**), respectively. **C** BHK-21 cells were infected with SVA or UV-inactivated SVA for 12 h and then processed and analysed as described in panel (**A**) **D** and **E** Proteins (**D**) and *RSAD2* mRNA (**E**) from PK-15 cells infected with SVA were analysed as described in panels **A** and (**B**) The data are expressed as the means ± standard deviations (SDs) from three independent experiments (*P < 0.05; **P < 0.01; ****P < 0.0001).

RSAD2, an ISG, plays an important role in the antiviral response [20]. To explore the relationship between SVA infection and RSAD2 expression, BHK-21 cells were infected with SVA and analysed at different time points by western blotting and RT-qPCR. Compared with mock infection, SVA infection reduced the protein expression and increased the transcript level of RSAD2, which peaked at 12 h post-infection (hpi) (Figures 1A and B). UV-inactivated SVA, which has no infectivity, did not affect RSAD2 expression (Figure 1C). Moreover, we analysed the changes in RSAD2 protein and mRNA levels in PK-15 cells and found that SVA infection triggered a decrease in RSAD2 protein levels and induced peak RSAD2 mRNA levels at 12 hpi, followed by a decrease (Figures 1D and E). These results indicated that only active SVA replication contributed to a reduction in RSAD2 expression levels.

### RSAD2 expression negatively regulates SVA replication

**Figure 2:**
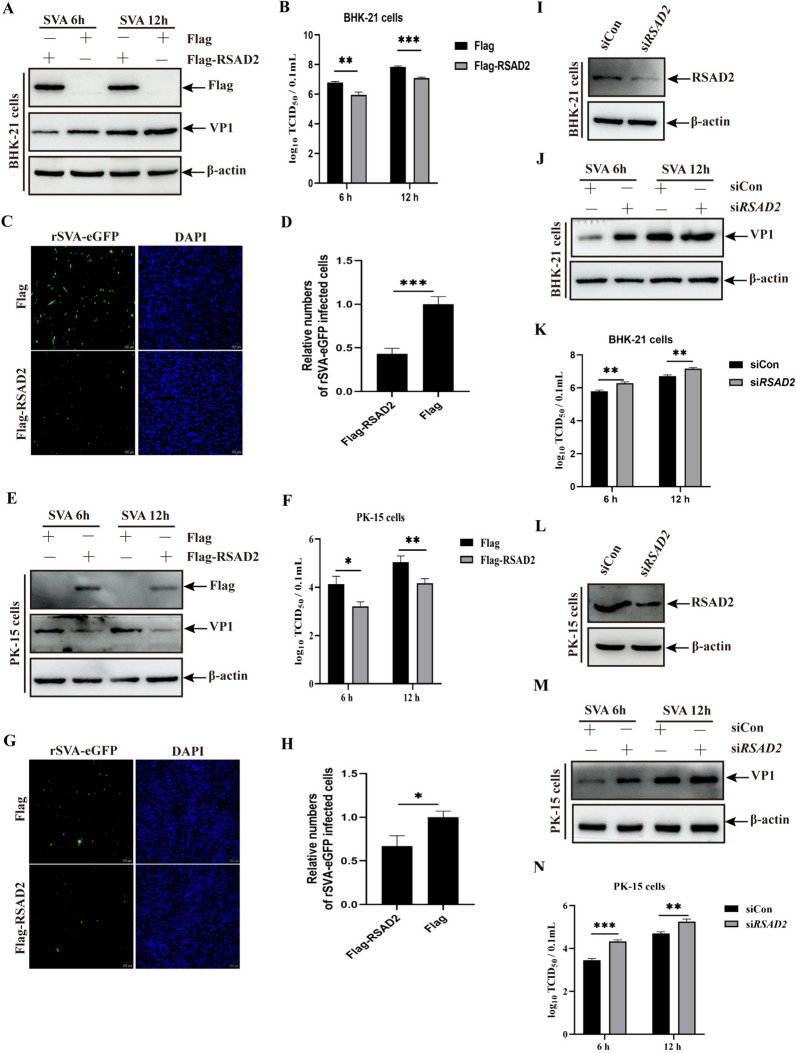
**RSAD2 inhibited SVA replication. A** and **B** BHK-21 cells transfected with Flag-RSAD2 or Flag plasmids were infected with SVA for 6–12 h, and the extracted proteins (**A**) and whole-cell culture medium (**B**) were then analysed. **C** and **D** BHK-21 cells transfected with Flag-RSAD2 or Flag plasmids were infected with rSVA-eGFP, and the number of rSVA-eGFP-infected cells was determined by an indirect fluorescence assay (IFA). **C**, and the results are presented in a histogram (**D**). **E** and **F** PK-15 cells transfected with Flag-RSAD2 or Flag plasmids were infected with SVA for 6–12 h, and the extracted proteins (**E**) and whole-cell culture media (**F**) were then analysed. **G** and **H** PK-15 cells transfected with Flag-RSAD2 or Flag plasmids were infected with rSVA-eGFP for 12 h, and the number of rSVA-eGFP-infected cells was then determined by IFA (**G**). The results are presented in a histogram (**H**). **I** and **L** Effects of RSAD2 silencing in BHK-21 (**I**) and PK-15 cells (**L**). **J**, **K**, **M**, and **N** BHK-21 and PK-15 cells transfected with si*RSAD2* or siCon were infected with SVA for 6–12 h, and the extracted proteins (J and M) and whole-cell culture media (K and N) were then analysed. The data are expressed as the means ± SDs from three independent experiments (*P < 0.05; **P < 0.01; ***P < 0.001).

To further explore the effect of RSAD2 on SVA replication, BHK-21 cells were transfected with Flag-RSAD2 and then infected with SVA. The western blotting results revealed that RSAD2 overexpression inhibited SVA replication at various time points (Figure 2A), and the viral titre results confirmed the antiviral effect of RSAD2 (Figure 2B). EGFP-tagged recombinant SVA (rSVA-eGFP) was used to analyse the effect of RSAD2 on viral infectivity in BHK-21 cells. The number of rSVA-eGFP-positive cells was significantly higher among mock-transfected cells than among RSAD2-expressing cells (Figures 2C and D). The inhibitory role of RSAD2 in SVA replication was analysed in PK-15 cells. As shown in Figures 2E–H, RSAD2 overexpression suppressed SVA replication, according to the results of viral VP1 expression, viral titre, and viral infectivity. Additionally, BHK-21 or PK-15 cells transfected with siRNA targeting *RSAD2* (si*RSAD2*) or siCon presented a substantial reduction in RSAD2 (Figures 2I and L), suggesting that si*RSAD2* successfully silenced RSAD2 expression. The effect of RSAD2 knockdown on SVA replication was further analysed. The results revealed that the silencing of RSAD2 increased VP1 expression and viral titres in BHK-21 and PK-15 cells at 6 and 12 hpi (Figures 2J, K, M, and N). These data indicate that RSAD2 plays an inhibitory role in SVA replication.

### RSAD2 interacts with SVA 2 C

**Figure 3:**
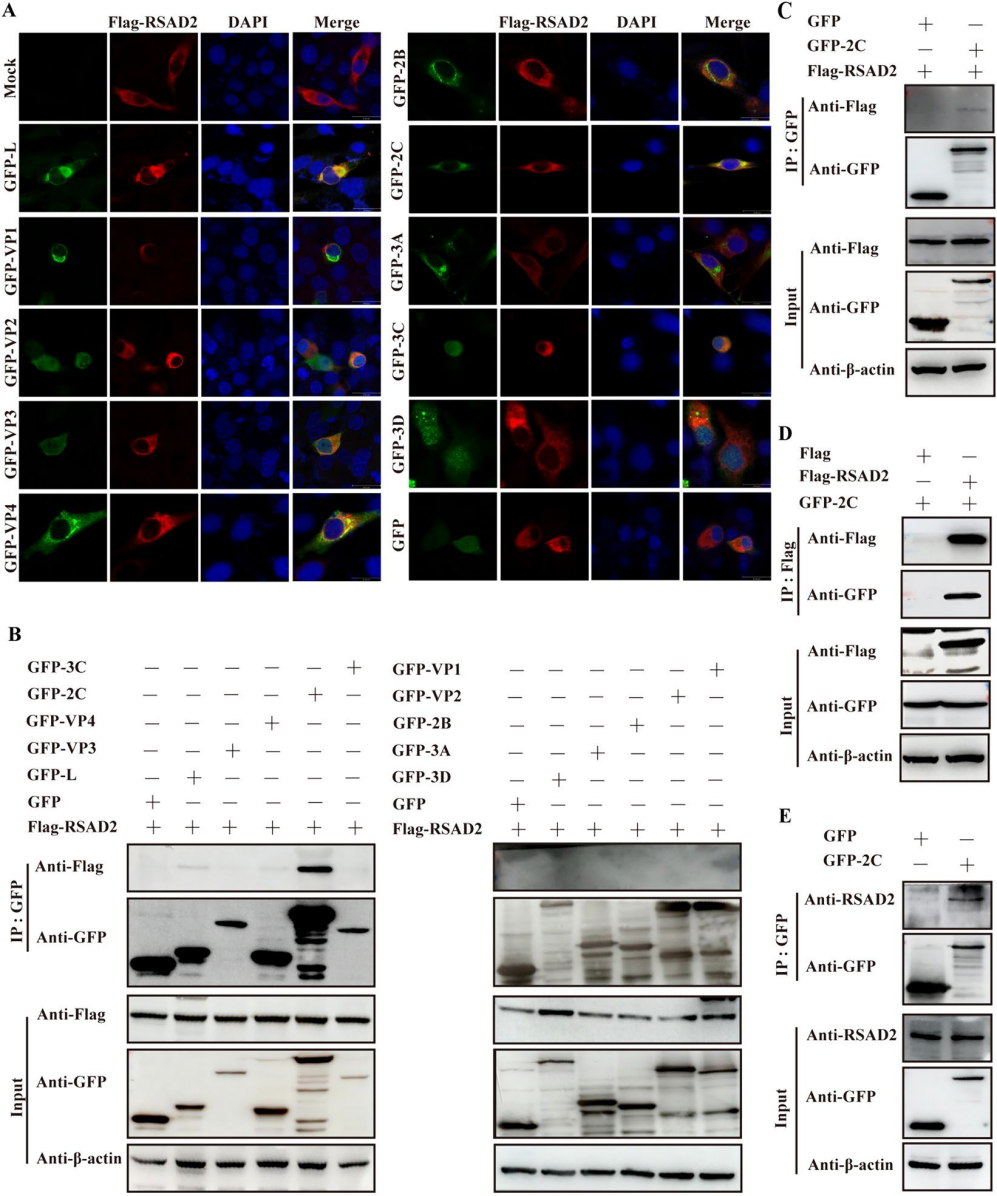
**RSAD2 interacted with SVA 2 C. A** BHK-21 cells were co-transfected with Flag-RSAD2 and various viral proteins linked with a GFP tag for 24 h, followed by fixation and incubation with anti-Flag antibodies (red signals) and DAPI (blue signals). The colocalization of RSAD2 and viral proteins was observed using confocal immunofluorescence microscopy. Scale bar, 20 μm. **B** HEK-293T cells co-expressing Flag-RSAD2 and GFP, GFP-L, GFP-VP1, GFP-VP2, GFP-VP3, GFP-VP4, GFP-2B, GFP-2 C, GFP-3 A, GFP-3 C, or GFP-3D for 24 h were lysed and analysed by co-IP. **C** and **D** HEK293T cells co-expressing GFP, GFP-2 C and/or Flag-RSAD2 for 24 h were lysed and immunoprecipitated with an anti-GFP or anti-Flag antibody, followed by co-IP analysis. **E** HEK**-**293T cells expressing GFP-2 C or GFP for 24 h were immunoprecipitated with an anti-GFP antibody, and endogenous RSAD2 was detected.

RSAD2 inhibits viral replication by interacting with viral proteins [19, 21]. It is assumed that the interaction between RSAD2 and viral proteins forms a basis for the regulation of viral replication by RSAD2. To identify SVA proteins that interact with RSAD2, we observed the colocalization of viral proteins and RSAD2 using confocal imaging. As shown in Figure 3A, RSAD2 was localized to SVA L, VP3, VP4, 2 C, and 3 C, indicating that these viral proteins may interact with RSAD2. To confirm the interaction between RSAD2 and these viral proteins, a coimmunoprecipitation (co-IP) assay was performed. A strong interaction between RSAD2 and SVA 2 C was observed, whereas weak bands in GFP-L- or GFP-VP4-expressing cells or no specific bands in other viral protein-expressing cells were observed (Figure 3B). The specific interaction between RSAD2 and SVA 2 C was subsequently validated via forward and reverse co-IP assays (Figure 3C and D). In cells expressing GFP-2 C or GFP alone, 2 C coprecipitated with endogenous RSAD2 (Figure 3E). These results reveal a specific interaction between RSAD2 and SVA 2 C.

### The aa 201–322 region of SVA 2 C is required for the interaction of RSAD2 and SVA 2 C

**Figure 4:**
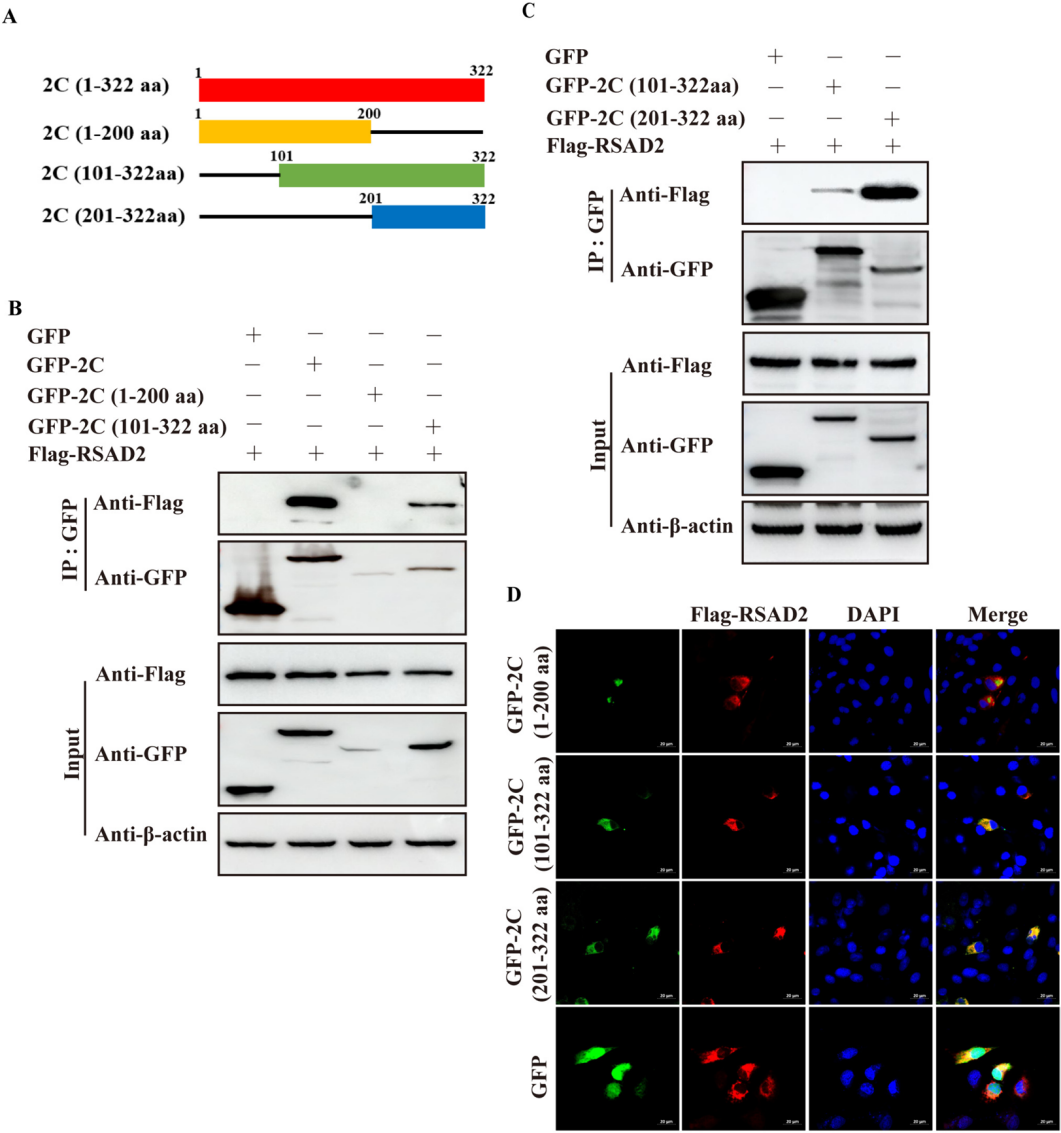
**The aa 201–322 domain of SVA 2 C plays an important role in the interaction between RSAD2 and SVA 2 C.A** Schematic representation of the lengths of various truncated SVA 2 C proteins. **B** HEK-293T cells co-expressing GFP, GFP-2 C, GFP-2 C (aa 1–200), or GFP-2 C (aa 201–322) and Flag-RSAD2 for 24 h were lysed, immunoprecipitated, and analysed via various antibodies. **C** HEK-293T cells co-expressing GFP, GFP-2 C (aa 101–322), or GFP-2 C (aa 201–200) and Flag-RSAD2 were analysed as described in panel A. **D** BHK-21 cells were co-transfected with pEGFP-C1, pEGFP-2 C (aa 1–200), pEGFP-2 C (aa 101–322), or pEGFP-2 C (aa 201–322) and Flag-RSAD2 plasmids and then processed as described in Figure 3A. Scale bar, 20 μm.

The full-length SVA 2 C protein was randomly divided into two segments (aa 1-200 and aa 101–322; Figure 4A), and the interaction between RSAD2 and the two segments of SVA 2 C was determined by co-IP. A specific band was detected in GFP-2 C (aa 101–322)-expressing cells, with GFP-2 C-expressing cells serving as the positive control group (Figure 4B). On the basis of these results, we inferred that the aa 201–322 domain of 2 C may be involved in the interaction between RSAD2 and 2 C (Figure 4A). We found that RSAD2 specifically interacted with GFP-2 C (aa 201–322), verifying this hypothesis (Figure 4C). Colocalization of RSAD2 and various 2 C segments was subsequently detected in BHK-21 cells. The results revealed that RSAD2 significantly colocalized with the aa 101–322 and aa 201–322 domains of 2 C (Figure 4D), suggesting that the interaction between RSAD2 and SVA 2 C was dependent mainly on the aa 201–322 domain of 2 C.

### The aa 43–70 region of RSAD2 plays an important role in SVA replication

**Figure 5:**
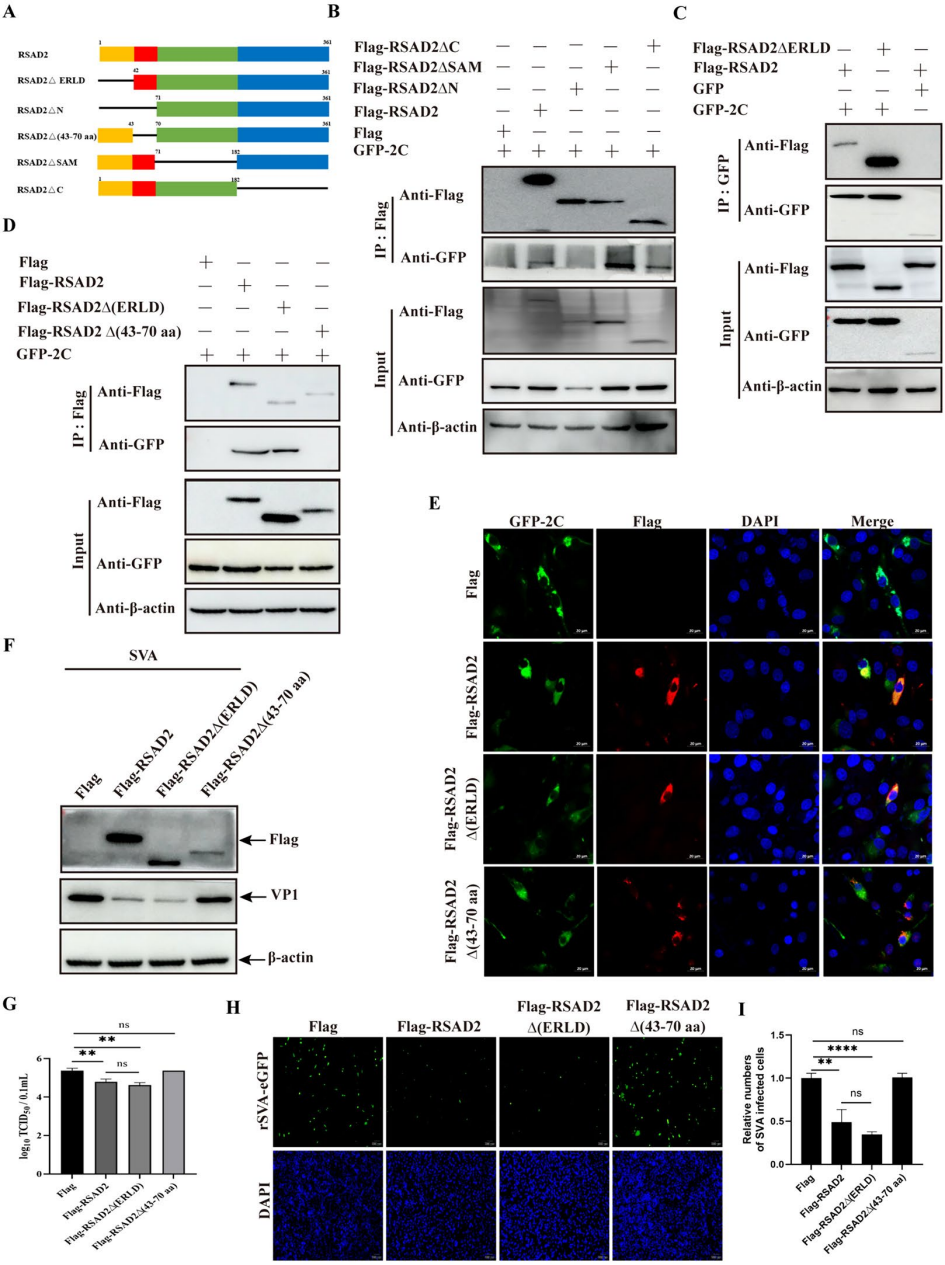
**The aa 43–70 region of RSAD2 is required for RSAD2-mediated inhibition of SVA replication.** **A** Schematic representation of various structural domains of RSAD2. **B** HEK-293T cells co-expressing Flag, Flag-RSAD2, Flag-RSAD2∆N, Flag-RSAD2∆SAM, or Flag-RSAD2∆C and GFP-2 C were lysed, immunoprecipitated with an anti-Flag antibody, and then analysed with various antibodies. **C** HEK-293T cells co-transfected with GFP or GFP-2 C and Flag-RSAD2 or Flag-RSAD2∆ERLD were processed, immunoprecipitated with an anti-GFP antibody, and then analysed as described in panel B. **D** HEK-293T cells co-transfected with GFP or GFP-2 C and Flag-RSAD2, Flag-RSAD2∆N, or Flag-RSAD2∆(aa 43–70) were processed, immunoprecipitated with an anti-GFP antibody, and then analysed as described in panel B. **E** BHK-21 cells were transfected as described in panel D, and the colocalization of SVA 2 C and RSAD2 mutants was observed. **F** and **G** BHK-21 cells transfected with Flag, Flag-RSAD2, Flag-RSAD2∆ (ERLD), or Flag-RSAD2∆ (aa 43–70) were infected with SVA for 12 h and then analysed for VP1 expression (**F**) and viral titres (**G**). **H** and **I** BHK-21 cells transfected with Flag-RSAD2 or its mutated plasmids were infected with rSVA-eGFP, and the number of rSVA-eGFP-infected cells was then determined by IFA (H). The results are presented in a histogram (**I**). The data are expressed as the means ± SDs from three independent experiments (ns, not significant; **P < 0.01; ****P < 0.0001).

RSAD2 contains an ER-localizing domain (aa 1–42), an N-terminal alpha-helical domain (aa 1–70), a central Fe-S cluster SAM domain (aa 71–182), and a highly conserved carboxy terminal (C-terminal) domain (aa 182–361; Figure 5A) [14]. On the basis of the structural domains of RSAD2, we constructed plasmids expressing different domains, such as Flag-RSAD2∆N, Flag-RSAD2∆C, Flag-RSAD2∆SAM, and Flag-RSAD2∆ER localizing domain (ERLD). To determine which domain of RSAD2 interacts with SVA 2 C, co-IP experiments were performed in plasmid-cotransfected cells. As shown in Figure 5B, only specific bands for RSAD2∆N and SVA 2 C disappeared in HEK-293T cells, suggesting that the N-terminus of RSAD plays an important role in the interaction between RSAD2 and SVA 2 C. Because the N-terminal domain of RSAD2 contains the ER-localizing domain (aa 1–42), the interaction region of RSAD2 and SVA 2 C was further analysed by co-IP. The results revealed the interaction of RSAD2∆ERLD and SVA 2 C (Figure 5C). Given the lack of interaction between RSAD2∆N and SVA 2 C, the aa 43–70 domain of RSAD2 may be responsible for the interaction between RSAD2 and SVA 2 C. The deletion of the aa 43–70 region of RSAD2 resulted in the loss of the ability of RSAD2 to interact and colocalize with SVA 2 C (Figure 5D and E). To explore the effects of the aa 43–70 region of RSAD2 on SVA replication, cells transfected with RSAD2, RSAD2∆N, or RSAD2∆ (aa 43–70) were infected with SVA and analysed by western blotting and viral titre assays. RSAD2 significantly inhibited SVA replication, as a positive control, whereas RSAD2∆N and RSAD2∆ (aa 43–70) reversed this inhibitory effect (Figure 5F and G). Additionally, we used rSVA-eGFP to further evaluate virus infectivity in cells transfected with RSAD2∆ (aa 43–70). The numbers of GFP-positive signals in the cells transfected with RSAD2∆ (aa 43–70) were similar to those in the cells transfected with RSAD2, both of which were significantly lower than the numbers among RSAD2∆ (ERLD)-expressing and control cells (Figure 5H and I), suggesting that the aa 43–70 domain of RSAD2 plays a crucial role in the inhibition of SVA replication by RSAD2.

### RSAD2 is involved in the antiviral effects of IFN-I in SVA-infected cells

**Figure 6:**
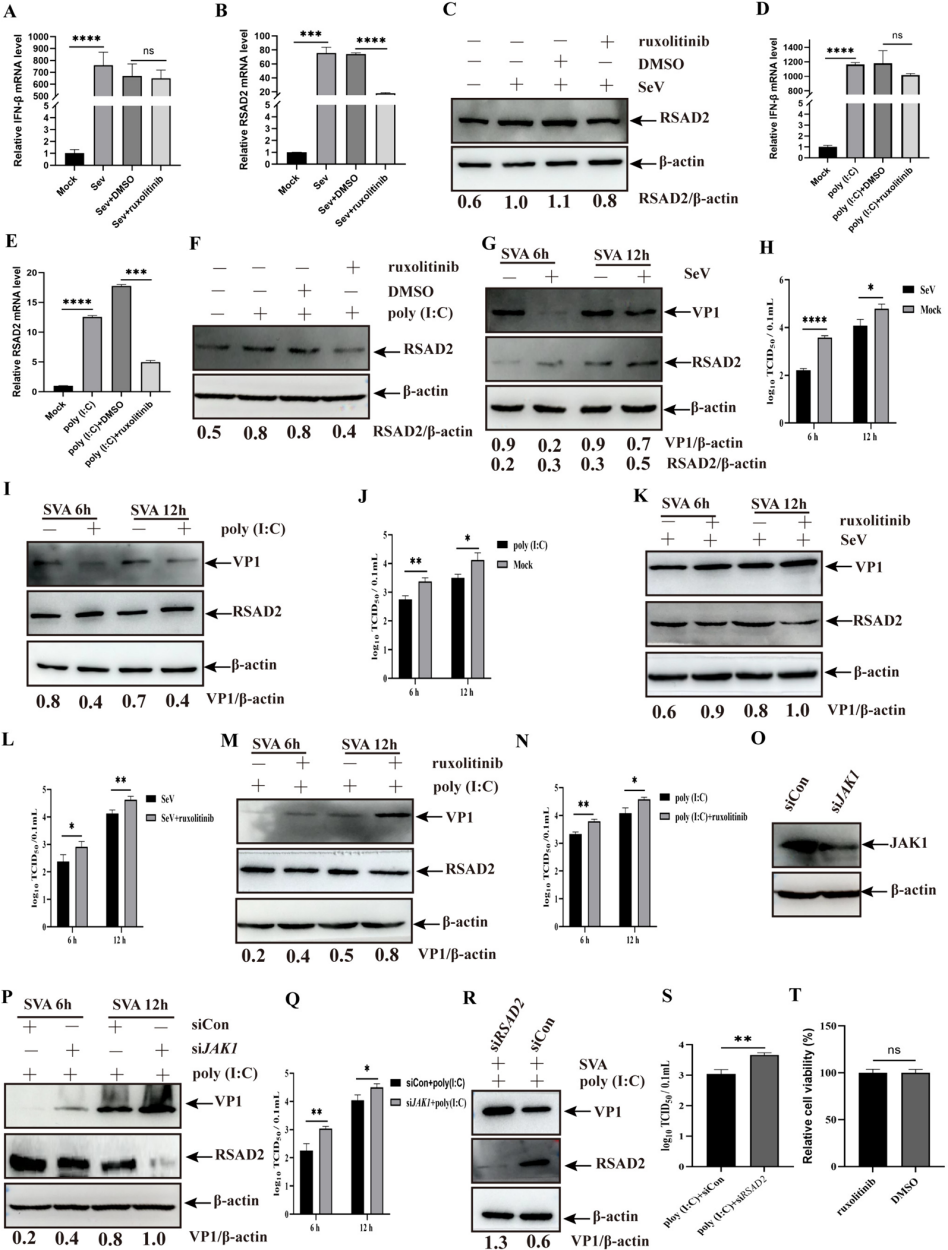
**Role of IFN-I in regulating RSAD2 expression in PK-15 cells. A-C** The levels of *IFN-β* (**A**) and *RSAD2* (**B**) mRNA or RSAD2 protein (**C**) were analysed by qPCR or western blotting, respectively, in PK-15 cells treated with SeV in the presence or absence of ruxolitinib. **D-F** The levels of *IFN-β* (**D**) and *RSAD2* (**E**) mRNA or RSAD2 protein (**F**) were analysed in poly (I: C)-transfected cells in the presence or absence of ruxolitinib. **G‒J** PK-15 cells treated with SeV or poly (I: C) were infected with SVA. The expression levels were then determined via anti-VP1, anti-RSAD2, or anti-β-actin antibodies (**G** and **I**), and the viral titres were subsequently calculated (**H** and **J**). **K–N** PK-15 cells treated with SeV or poly (I: C) were infected with SVA, followed by treatment with ruxolitinib. The expression levels were then determined via anti-VP1, anti-RSAD2, or anti-β-actin antibodies (**K** and **M**), and viral titres were calculated (**L** and **N**). **O** Effect of JAK1 silencing in PK-15 cells (**O**). **P** and **Q** PK-15 cells transfected with si*JAK1* or siCon and treated with poly (I: C) were infected with SVA for 6–12 h, and the extracted proteins (**P**) and whole-cell culture media (**Q**) were then analysed. **R** and **S** PK-15 cells were transfected with siRSAD2 or siCon and treated with poly (I: C), followed by infection with SVA for 12 h and analysis as described in panels R and S. Viability assay in PK-15 cells treated or not treated with ruxolitinib (1 µM). T The detection of cell viability in ruxolitinib-treated and-untreated PK-15 cells. The data are expressed as the means ± SDs from three independent experiments (ns, not significant [*P* > 0.05]; *P < 0.05; **P < 0.01; ***P < 0.001; ****P < 0.0001).

RSAD2 is upregulated by viral infection or IFN-I/II signalling through the Janus kinase signal transducer and activator of transcription (JAK-STAT) pathway [20]. To verify the regulatory role of IFN in RSAD2 expression, PK-15 cells were treated with two IFN stimulators, SeV and poly (I: C). The results revealed that the levels of *IFN-β* and *RSAD2* mRNA and RSAD2 expression substantially increased (Figure 6A–F). To further explore whether the increase in RSAD2 expression was mediated by IFN-β, ruxolitinib, an IFN-β downstream signal molecule (JAK) inhibitor, was added to the cells treated with SeV or poly (I: C). Ruxolitinib treatment did not affect the increase in *IFN-β* mRNA levels (Figure 6A and D) but significantly reduced *RSAD2* mRNA levels (Figure 6B and E). Moreover, the reduction in RSAD2 expression caused by ruxolitinib treatment confirmed these results (Figure 6C and F), suggesting that JAK plays an important role in the IFN-β-induced increase in RSAD2 expression.

Activation of the innate immune pathway following infection with an RNA virus or double-stranded RNA (dsRNA) induces the production of IFN-I and IFN-II and then mediates ISGs [22, 23]. First, we analysed the effects of SeV or poly (I: C), which are IFN-I inducers, on SVA replication. PK-15 cells treated with SeV or poly (I: C) were infected with SVA, after which the change in SVA replication was analysed. Compared with control treatment, SeV infection or poly (I: C) treatment substantially reduced the VP1 expression level and viral titre (Figures 6G–J). To explore the role of RSAD2 in the inhibition of SVA replication by SeV infection or poly (I: C) treatment, PK-15 cells were treated with ruxolitinib and infected with SVA. As shown in Figures 6K–N, xolitinib treatment significantly weakened the inhibitory effects of SeV and poly(I: C) on SVA replication. To exclude the nonspecific role of chemical reagents in viral replication, siRNA targeting *JAK1* (si*JAK1*) was used to analyse the inhibitory effect of poly (I: C) on viral replication. Silencing of JAK1 via si*JAK1* attenuated poly (I: C)-induced anti-SVA activity (Figures 6O–Q). Subsequently, PK-15 cells transfected with si*RSAD2* were infected with SVA, and SVA replication was subsequently analysed. The silencing of *RSAD2* significantly disrupted the inhibitory effects of poly (I: C) on SVA replication (Figures 6R and S). To exclude the cytotoxicity of ruxolitinib, cell viability was measured using MTT assays. The cell viability was not different from that of the ruxolitinib-untreated group (T). These data revealed that RSAD2 plays a crucial role in the IFN-I-mediated inhibition of SVA replication.

## Discussion

Infection with different viruses leads to differences in RSAD2 expression levels. Some viruses induce the upregulation of both RSAD2 mRNA and protein expression during infection, but their antiviral activity is impaired. For example, EV71 or CSFV infection significantly increases RSAD2 mRNA and protein levels [19, 21], which differs from the effects of SVA infection. In our study, the mRNA level of *RSAD2* significantly increased, whereas its protein expression level decreased during SVA infection (Figure 1). Additionally, the HSV-1 UL41 protein markedly abrogates the antiviral activity of RSAD2 by reducing its mRNA expression level [24].

RNA viral infection activates the innate immune system to produce IFN and its downstream products, ISGs [22, 23]. RSAD2 is an important ISG that mediates broad antiviral activity against various viruses by impairing viral proliferation processes, including viral adsorption, entry, replication, and release [25]. Our results revealed that the overexpression and silencing of RSAD2 significantly inhibited and promoted SVA replication and infection at various time points in PK-15 and BHK-21 cells, respectively (Figure 2), suggesting that the anti-SVA activity of RSAD2 has universality rather than specificity in different cells. Importantly, the above results also directly demonstrated that the anti-SVA mechanism of RSAD2 was independent of the upstream IFN pathway on the basis of the inhibitory effect of RSAD2 on SVA replication in BHK-21 cells (IFN-deficient cells). The antiviral function of RSAD2 is related to host factors. For example, RSAD2 expression regulates the formation of lipid rafts, which affect plasma membrane fluidity and disrupt influenza virus budding by interacting with farnesyl diphosphate synthase [26]. RSAD2-mediated antiviral activity is enhanced by its interaction with and inhibition of Golgi brefeldin A-resistant guanine nucleotide exchange factor 1, which blocks the assembly of tick-borne encephalitis virus (TBEV) particles and the release of malfunctioning-membrane-associated capsid particles [27]. In addition to altering the function of host proteins, RSAD2 also inhibits viral replication by interacting with host proteins. The mitochondrial translocation of rotavirus NSP4 is impeded by RSAD2, which reduces the cytosolic release of cytochrome c and inhibits mitochondrial apoptosis [28]. RSAD2 restricts Zika virus and TBEV replication by targeting NS3 for proteasomal degradation [27]. Similarly, our results showed that RSAD2 has a negative regulatory effect on SVA replication by specifically interacting with the SVA 2 C protein (Figure 3), prompting us to explore its inhibitory mechanism on the basis of the distinct domains of RSAD2.

RSAD2 consists of an N-terminal-domain-containing ER localization region, an intermediate SAM domain, and a C-terminal domain [14]. The different structural domains of RSAD2 exhibit diverse antiviral mechanisms. The N-terminal domain of RSAD2 is crucial for its anti-chikungunya virus activity, which depends on its localization in the ER [29]. RSAD2, through its radical SAM activity, depletes cellular nucleotide pools and interferes with mitochondrial metabolism, inhibiting viral replication [30]. Early dengue virus type-2 RNA production/accumulation is restricted by the C-terminal domain of RSAD2 via interactions with viral NS3 and the replication complex [25]. However, the effects of specific RSAD2 domains on SVA replication have not been elucidated. Our results revealed that only the N-terminal region of RSAD2 was involved in the interaction with the SVA 2 C protein, whereas aa 43–70 of RSAD2, instead of its ER localization domain (aa 1–42), was responsible for this interaction, and the deletion of this interaction region eliminated the anti-SVA activity of RSAD2 (Figure 5), indicating that RSAD2 aa 43–70 is a key region for inhibiting SVA replication by interacting with the viral 2 C protein. These results confirmed that the anti-SVA mechanism of RSAD2 is independent of the upstream IFN signalling pathway. Similarly, the RSAD2 N-terminal domain plays an important role in interaction with the EV71 2 C protein and in the suppression of viral replication [31]. However, more precise functional regions were not explored in this study. The ER participates in the assembly of replication complexes and provides a platform for viral replication, budding, and release using membranes derived from the ER [32]. Moreover, viral 2 C proteins are involved in the formation of replication complexes during picornaviral infection [33]. Thus, we speculate that the antiviral activity of RSAD2 mediated by its aa 43–70 region may be associated with impairment of the viral replication complex after its localization to the ER via its ER localization domain.

IFN levels are increased by SeV infection, and poly (I: C) binds to IFN receptors and initiates the JAK-STAT pathway, producing a subset of ISGs with antiviral activities [34, 35]. Ruxolitinib, a JAK-STAT pathway inhibitor, reduces the expression levels of ISGs, such as RSAD2 [36]. The replication of various viruses inhibited by IFN prompted us to explore whether RSAD2, an ISG, is involved in IFN-mediated antiviral activity. In our study, SeV infection- or poly (I: C) treatment-induced RSAD2 expression was inhibited by ruxolitinib, si*JAK1*, or si*RSAD2*, disrupting their inhibitory effect on PK-15 cells (Figure 6). These results indicate that RSAD2, a molecule downstream of IFN, is involved in anti-SVA activity.

In conclusion, we demonstrated that SVA infection blocks the antiviral activity of RSAD2 by reducing RSAD2 expression. The mechanism by which RSAD2 suppresses SVA replication is dependent mainly on the direct interaction between the RSAD2 aa 43–70 region and the SVA 2 C protein. Further results confirmed that RSAD2, an ISG, plays an important role in the IFN-mediated anti-SVA effect in PK-15 cells. Clarifying the inhibitory mechanism of RSAD2 will contribute to a better understanding of viral pathogenesis.
